# Understanding maternity care provision and experience for autistic women and birthing people and their care providers in the UK: protocol for a scoping review

**DOI:** 10.1136/bmjopen-2025-114558

**Published:** 2026-07-28

**Authors:** Joanne Welsh, Kathryn Redmond, Joanne McPeake, Nik Nikolić, Ann-Beth Moller

**Affiliations:** 1 Cambridge University Hospitals NHS Foundation Trust, Cambridge, UK; 2 University of Cambridge, Cambridge, UK; 3 Department of Public Health and Primary Care, The Healthcare Improvement Studies Institute, University of Cambridge, Cambridge, UK; 4 Clinical Neuroscience Department, Brighton & Sussex Medical School, University of Sussex, Brighton, UK; 5 School of Public Health and Community Medicine, Institute of Medicine, Sahlgrenska Academy, University of Gothenburg, Gothenburg, Sweden

**Keywords:** Midwifery, Health Services Accessibility, Quality in health care, Mothers, Patient-Centered Care

## Abstract

**Abstract:**

**Introduction:**

Maternity care in the UK is under increasing scrutiny, with reports indicating that services often fail to meet the diverse needs of women and birthing people. Autism spectrum disorder is a neurodevelopmental condition involving persistent difficulties in social communication and interaction alongside restricted and repetitive behaviours. Autistic women and birthing people may face distinct barriers to accessing and navigating maternity care, compounded by limited awareness and inflexible service provision. To improve care responsiveness, it is essential to understand both the challenges faced by autistic women and birthing people and those encountered by healthcare professionals in delivering optimal care. This scoping review aims to identify and map existing literature and evidence on maternity care in the UK for autistic women and birthing people.

**Methods:**

A comprehensive search, restricted to January 2000 to July 2026, will be conducted across multiple databases, including the Cumulative Index of Nursing and Allied Health Literature, PubMed/MEDLINE, Embase and the Overton Index. Reference lists of included studies will be screened to identify additional relevant literature. Extracted data will include study context, research focus and key findings.

**Analysis:**

The literature will be mapped across several domains: experiences of care among autistic women and birthing people; maternity care providers’ knowledge of autism and ability to provide optimal care for autistic women and birthing people; interventions designed to support this population and relevant government policies influencing practice.

**Ethics and dissemination:**

As this review involves secondary analysis of published literature, ethical approval is not required. Findings will be presented using tabular and diagrammatic formats, supported by narrative synthesis. Results will be disseminated through publication in a peer-reviewed journal and via relevant professional networks and charity partners, and may inform future research, policy and practice in maternity care.

**Strengths and limitations of this study**

- The review uses a systematic and transparent methodology, following established scoping review frameworks, enhancing the reliability and reproducibility of the findings.
- A potential limitation is that the review may miss relevant research that has not been published in peer-reviewed journals.
- The review does not assess the quality of evidence, as is standard for scoping reviews, which may affect interpretation of findings.

## Introduction

 Pregnancy, childbirth and the transition to motherhood are significant life events for women and birthing people^1^ (see box 1 for how we define women and birthing people for the purpose of this review). These experiences can be both physically and emotionally demanding, underscoring the need for high-quality maternity care that ensures psychological and physical safety.^2^ Such care is essential not only for the well-being of the mother and baby but also for supporting a positive transition into parenthood.

Box 1 **Definition of women and birthing people**

We use the term women and birthing people for all individuals who are pregnant and give birth, including cisgender women, transgender men, non-binary people and others whose gender identity may not align with the traditional category of ‘woman’.

Recent inquiries into maternity services in the UK—including the Morecambe Bay Investigation,^3^ the Independent Maternity Review^4^ and the East Kent Investigation^5^—have culminated in the commissioning of a National Investigation into maternity care. Alongside the Birth Trauma Report,^6^ findings from each of these reports highlight systemic failures in meeting the needs of those accessing maternity services. For some, particularly those facing health inequities, these shortcomings are often further compounded.^6 7^ Autistic women and birthing people are one such group.

Autism spectrum disorder (ASD) as defined in the International Classification of Diseases 11th Revision (ICD-11),^8^ see box 2 for full definition, is estimated to affect 1 in 100 people in the UK.^9^ Autism is understood as a spectrum, with considerable variation in how it presents from person to person. Notably, in some cases, especially in women, people can mask their autism. Thus, an estimated 80% of autistic women are undiagnosed by age 18.^10^ Autistic individuals often experience co-occurring physical and mental health conditions^11 12^ with a higher prevalence of mental health challenges compared with non-autistic populations.^13^ Rong *et al* (2021) estimate co-occurring rates of Attention Deficit Hyperactivity Disorder (ADHD) in individuals with ASD to be 38.5%.^14^ For autistic women, these factors may contribute to barriers in accessing and receiving quality maternity care.

Box 2 **Definition of autism spectrum disorder (6 A02) according to the International Classification of Diseases 11th Revision (ICD-11)^8^**

Autism spectrum disorder is characterised by persistent deficits in the ability to initiate and to sustain reciprocal social interaction and social communication, and by a range of restricted, repetitive and inflexible patterns of behaviour, interests or activities that are clearly atypical or excessive for the individual’s age and sociocultural context. The onset of the disorder occurs during the developmental period, typically in early childhood, but symptoms may not become fully manifest until later, when social demands exceed limited capacities. Deficits are sufficiently severe to cause impairment in personal, family, social, educational, occupational or other important areas of functioning and are usually a pervasive feature of the individual’s functioning observable in all settings, although they may vary according to social, educational or other context. Individuals along the spectrum exhibit a full range of intellectual functioning and language abilities.

Pregnancy can introduce a range of sensory challenges, including nausea, heightened sensitivity to smell and taste, fetal movements, physical examinations and labour pain.^15^ These experiences may trigger hyper- or hypo-sensitivities.^16^ Differences in communication style and information processing^17–19^ can further complicate interactions with maternity care providers, potentially limiting autistic individuals’ ability to express their needs. The hospital environment—characterised by bright lights, unfamiliar people and busy clinical settings—can be overwhelming and anxiety-inducing,^17 18^ sometimes leading to disengagement from care.^16^ Additionally, a lack of autism-specific training and awareness among healthcare professionals may hinder the provision of responsive and inclusive care.^20 21^ This can lead to poor outcomes, for example, evidence from Sweden has reported a higher incidence of medically indicated preterm birth, elective caesarean birth and pre-eclampsia in this group compared with non-autistic women and birthing people.^22^

While previous reviews have explored autistic women and birthing people’s experiences of maternity care, none have comprehensively examined the broader landscape—including national guidance, healthcare professionals’ knowledge of autism and ability to provide optimal care for autistic women and birthing people, and targeted interventions. This scoping review aims to address this gap by identifying and mapping existing literature related to the experiences of maternity care for autistic women in the UK, healthcare professionals’ knowledge of autism and their experience of providing care for autistic women as well as relevant government policies influencing practice.

## Methods and analysis

### Patient and public involvement

No patients or members of the public were included in the design of this review. However, findings from the review will be shared with patient and public involvement and engagement groups to support the design of dissemination activities and implementation of learning.

### Study design

Due to the nature of the research question, a scoping review was chosen to support the identification of all literature relevant to maternity care for autistic women and birthing people in the UK. A scoping review allows the exploration of the evidence available on the topic.^23^ This is opposed to a systematic review which is more suitable to examine questions related to feasibility, effectiveness or appropriateness of a particular treatment or practice—none of which pertain to this particular study.^23^ This scoping review follows the steps outlined by the Joanna Briggs Institute (JBI) methodology for scoping reviews.^24^ The Preferred Reporting Items for Systematic reviews and Meta-Analyses extension for Scoping Reviews (PRISMA-ScR) checklist^25^ (see online supplemental file 1) will be used to present the findings of the scoping review.

### Research questions

The objective of this study is to understand maternity care provision and experience for autistic women and birthing people and their care providers in the UK. It aims to achieve this by addressing the following questions:

1. What are the experiences of autistic women and birthing people when accessing maternity care in the UK?
2. What knowledge do healthcare professionals providing maternity care have about the effect of autism on the provision of care for autistic women and birthing people?
3. How do healthcare professionals find the experience of providing maternity care for autistic women and birthing people in the UK?
4. What national policies and clinical guidelines exist to support maternity care for autistic women and birthing people in the UK?

### Inclusion criteria

Relevant qualitative and quantitative literature will be selected specific to the Population, Concept, Context criteria outlined in table 1. The design and delivery of maternity care differ from country to country with marked differences seen in the provision of care in high-income countries compared with low-income and middle-income countries.^26^ However, this is not to say care provision is similar in all high-income countries. For example, in the USA, care provision can be fragmented, with significant differences in access and quality depending on insurance status and geographic location.^27^ In Australia, while public maternity care is widely available, indigenous and remote communities often experience barriers to equitable care.^28^ Furthermore, the UK is experiencing its own challenges in terms of the delivery of maternity care as highlighted in several recent reviews.^3–5^ The study will therefore focus on UK maternity care for autistic women and birthing people, and only studies relevant to the UK context and grey literature pertaining to the UK will be included in the review.

**Table 1:** Inclusion criteria for identifying eligible studies

	Inclusion criteria
Population	Autistic women and birthing people who have accessed maternity care at any time during the pregnancy, birth and postnatal period. Healthcare professionals involved in providing maternity care to autistic women and birthing people. This may include but is not limited to midwives, maternity care assistants, obstetricians, anaesthetists, neonatologists and mental health workers.
Concept	Understand pertinent issues related to maternity care for autistic women and birthing people, including autistic women’s experience of receiving care, and knowledge maternity care providers have about the effect of autism on the provision of maternity care for autistic women and their experience of delivering this care, as well as local and national guidance shaping practice.
Context	Maternity care provision for autistic women and birthing people in the UK.

### Exclusion criteria

Articles will not be eligible for inclusion in the review if:

1. Autism is not the primary diagnosis. For example, if a study reports on women and birthing people with ASD and ADHD, the study will be included. However, if a study reports on women and birthing people with ADHD only, it will not be included.
2. The country in which the study has taken place is not the UK.
3. The publication is not in the English language.
4. The healthcare providers taking part in the study are not considered to be providers of maternity care.

### Search strategy

An initial search of Medline was undertaken to identify literature on the topic and to test search terms and key words. Details of this search, including the search strategy can be found in online supplemental file 2. From this, search terms will be refined following consultation with a research librarian and adapted for the following databases Nursing and Allied Health Literature (CINAHL), PubMed/MEDLINE and Embase. The reference list of all eligible studies will be screened to identify any additional studies. Searches will be restricted to English language and to the time frame January 2000 to July 2026 to capture evidence over the past 25 years. Overton Index (https://www.overton.io) is the world’s largest searchable database of policy documents and grey literature, designed to show how research influences policy and where policymakers are asking for evidence. Overton Index will be used for searches of policy documents and grey literature related to maternity care for autistic women and birthing people.

### Study selection

All identified records will be uploaded to Zotero (Digital Scholar—https://www.zotero.org), a reference management software application, where duplicates will be removed. Results will then be imported into Covidence (https://www.covidence.org/) for title and abstract screening. Studies will be screened independently by three reviewers (JW, A-BM and KR). Following screening of the first 100 articles, the three reviewers will meet to discuss inconsistencies. A fourth reviewer will be consulted in the event of any unresolvable disagreements. Articles identified for inclusion will have their full texts imported into Covidence, and will be independently screened by JW, A-BM and KR. Any articles excluded at this stage will have reasons for exclusion documented.

### Data extraction and analysis

A scoping review aims to provide an overview of the body of evidence available on a topic. Results of the literature search, in terms of number of records identified, and included and excluded records, including reasons for exclusion, will be mapped out using the PRISMA flow diagram^29^ (see online supplemental file 3).

A data collection tool (see online supplemental file 4) will be used to extract data. Data will be extracted independently by three reviewers (JW, A-BM and KR). Following extraction, the three reviewers will meet to discuss inconsistencies. A fourth reviewer will be consulted in the event of any unresolvable disagreements. Information retrieved will include details about the study, year, study design, context and main findings. Main findings extracted will include key themes related to autistic women’s experiences of maternity care, knowledge of maternity care providers about autism and their experience of providing care and key recommendations for the provision of care of autistic women. The data collection tool will be piloted on two papers, following the process described above, that are included following the search, and may be modified during the data extraction process to allow for flexibility should any other key information need to be included. Findings will be presented using tabular and diagrammatic formats, supported by a narrative summary that will discuss results and link them to the objectives of the review.

## Ethics and dissemination

This scoping review involves secondary analysis of publicly available literature and does not include human participants or identifiable personal data. Therefore, ethical approval is not required. The review will follow established methodological frameworks to ensure transparency and rigour.

Findings will be disseminated through publication in a peer-reviewed journal, targeting audiences in maternal health, neurodiversity and health services research. Where relevant, findings will be shared as conference presentations and at stakeholder meetings to engage practitioners, policymakers and advocacy groups.

## Supplementary material

10.1136/bmjopen-2025-114558 online supplemental file 1

10.1136/bmjopen-2025-114558 online supplemental file 2

10.1136/bmjopen-2025-114558 online supplemental file 3

10.1136/bmjopen-2025-114558 online supplemental file 4

## References

[1] Grant AD, Erickson EN (2022). Birth, love, and fear: Physiological networks from pregnancy to parenthood. Compr Psychoneuroendocrinol.

[2] O’Reilly E, Buchanan K, Bayes S (2025). Emotional safety in maternity care: An evolutionary concept analysis. Midwifery.

[3] Kirkup B (2015). The report of the morecambe bay investigation.

[4] Independent Maternity Review (2022). Ockenden Report – Final: Findings, Conclusions, and Essential Actions from the Independent Review of Maternity Services at the Shrewsbury and Telford Hospital NHS Trust.

[5] Kirkup B (2022). Reading the Signals: Maternity and Neonatal Services in East Kent – the Report of the Independent Investigation.

[6] All-Party Parliamentary Group on Birth Trauma (2024). Listen to mums: ending the postcode lottery on perinatal care.

[7] Felker A, Patel R, Kotnis R (2025). Saving Lives, Improving Mothers’ Care: Lessons Learned to Inform Maternity Care from the UK and Ireland Confidential Enquiries into Maternal Deaths and Morbidity 2021-23.

[8] World Health Organization International classification of diseases 11th revision (icd-11) Geneva. https://icd.who.int/en.

[9] National Autistic Society (2025). What is autism?. https://www.autism.org.uk/advice-and-guidance/what-is-autism.

[10] McCrossin R (2022). Finding the True Number of Females with Autistic Spectrum Disorder by Estimating the Biases in Initial Recognition and Clinical Diagnosis. Children (Basel).

[11] Ingudomnukul E, Baron-Cohen S, Wheelwright S (2007). Elevated rates of testosterone-related disorders in women with autism spectrum conditions. Horm Behav.

[12] Simantov T, Pohl A, Tsompanidis A (2022). Medical symptoms and conditions in autistic women. Autism.

[13] Lai M-C, Kassee C, Besney R (2019). Prevalence of co-occurring mental health diagnoses in the autism population: a systematic review and meta-analysis. Lancet Psychiatry.

[14] Rong Y, Yang C-J, Jin Y (2021). Prevalence of attention-deficit/hyperactivity disorder in individuals with autism spectrum disorder: A meta-analysis. Res Autism Spectr Disord.

[15] Westgate V, Sewell O, Caramaschi D (2024). Autistic Women’s Experiences of the Perinatal Period: A Systematic Mixed Methods Review. Rev J Autism Dev Disord.

[16] Fox D (2022). Exploring how health inequalities can be addressed through autism training and understanding in maternity services.

[17] Moore L, Foley S, Larkin F (2025). Understanding the experiences of receiving and providing maternity care for autistic adults: A Multi-perspectival Interpretative Phenomenological Analysis study. Autism.

[18] Samuel P, Yew RY, Hooley M (2022). Sensory challenges experienced by autistic women during pregnancy and childbirth: a systematic review. Arch Gynecol Obstet.

[19] Nicolaidis C, Raymaker DM, Ashkenazy E (2015). “Respect the way I need to communicate with you”: Healthcare experiences of adults on the autism spectrum. Autism.

[20] King S (2023). How Do Maternity Services Support Autistic Women and Birthing People Now? What Improvements Could Be Made to Help Autistic People Who Are Pregnant and Giving Birth and the Staff Who Support Them?.

[21] Maddox BB, Crabbe S, Beidas RS (2020). “I wouldn’t know where to start”: Perspectives from clinicians, agency leaders, and autistic adults on improving community mental health services for autistic adults. Autism.

[22] Sundelin HE, Stephansson O, Hultman CM (2018). Pregnancy outcomes in women with autism: a nationwide population-based cohort study. Clin Epidemiol.

[23] Pollock D, Davies EL, Peters MDJ (2021). Undertaking a scoping review: A practical guide for nursing and midwifery students, clinicians, researchers, and academics. J Adv Nurs.

[24] Peters MDJ, Marnie C, Tricco AC (2020). Updated methodological guidance for the conduct of scoping reviews. JBI Evid Synth.

[25] Tricco AC, Lillie E, Zarin W (2018). PRISMA Extension for Scoping Reviews (PRISMA-ScR): Checklist and Explanation. Ann Intern Med.

[26] United Nations Population Fund (2021). State of the world’s midwifery 2021 (SoWMy 2021).

[27] Sonenberg A, Mason DJ (2023). Maternity Care Deserts in the US. JAMA Health Forum.

[28] McLachlan H, Newton M, McLardie-Hore F (2023). Improving outcomes for First Nations mothers and babies in Australia through culturally safe continuity of midwifery care: the time for scale-up is now!. EClinicalMedicine.

[29] Page MJ, McKenzie JE, Bossuyt PM (2021). The PRISMA 2020 statement: an updated guideline for reporting systematic reviews. BMJ.

