# Supplementary material for: Understanding maternity care provision and experience for autistic women and birthing people and their care providers in the UK: protocol for a scoping review
Source: BMJ Open. 2026 Jul 28;16(7):e114558. doi: 10.1136/bmjopen-2025-114558 (PMC13423141; doi:10.1136/bmjopen-2025-114558)
Supplement: online supplemental file 2 [file bmjopen-16-7-s002.pdf]

Supplementary File 2. Test search strategies

Conducted in Medline and Ovid

Ovid MEDLINE(R) ALL <1946 to August 11, 2025>
https://ovidsp.ovid.com/athens/ovidweb.cgi?T=JS&NEWS=N&PAGE=main&SHAREDSEARCHID=12qpzLftZl47fmHV5RcA0voWp6jtQxXCt1DJ3PVYjZPRwSKU4yCzG8sGR6phO2AFS

1 exp Autism Spectrum Disorder/ 49294

2 (autis* or asperger* or asd or "autism spectrum disorder" or neurodivergen*).ti,ab. 89369

3 1 or 2 92751

4 (pregna* or maternity or prenatal or perinatal or postnatal or postpartum or birth or delivery or labour or labor or "pregnancy outcome").ti,ab. 1722697

5 exp Pregnancy/ or exp Maternal Health Services/ or Hospitals, Maternity/ 1075758

6 exp "Obstetrics and Gynecology Department, Hospital"/ or exp Obstetrics/ 28127

7 obstet*.ti,ab. 127011

8 (midwif* or midwiv*).ti,ab. 32177

9 exp Midwifery/ 22539

10 4 or 5 or 6 or 7 or 8 or 9 2164581

11 (experienc* or support or care or perception* or perspective* or reflection* or guideline* or best practice* or "code of practice").ti,ab. or exp patient satisfaction/ 5577862

12 3 and 10 and 11 2332

13 exp United Kingdom/ 406682

14 (national health service* or nhs*).ti,ab,in. 320811

15 (english not ((published or publication* or translat* or written or language* or speak* or literature or citation*) adj5 english)).ti,ab. 148315

16 (gb or "g.b." or britain* or (british* not "british columbia") or uk or "u.k." or united kingdom* or (england* not "new england") or northern ireland* or northern irish* or scotland* or scottish* or ((wales or "south wales") not "new south wales") or welsh*).ti,ab,jw,in. 2701845

17 (bath or "bath's" or ((birmingham not alabama*) or ("birmingham's" not alabama*) or bradford or "bradford's" or brighton or "brighton's" or bristol or "bristol's" or

carlisle* or "carlisle's" or (cambridge not (massachusetts* or boston* or harvard*)) or ("cambridge's" not (massachusetts* or boston* or harvard*)) or (canterbury not zealand*) or ("canterbury's" not zealand*) or chelmsford or "chelmsford's" or chester or "chester's" or chichester or "chichester's" or coventry or "coventry's" or derby or "derby's" or (durham not (carolina* or nc)) or ("durham's" not (carolina* or nc)) or ely or "ely's" or exeter or "exeter's"

or gloucester or "gloucester's" or hereford or "hereford's" or hull or "hull's" or lancaster or "lancaster's" or leeds* or leicester or "leicester's" or (lincoln not nebraska*) or ("lincoln's" not nebraska*) or (liverpool not (new south wales* or nsw)) or ("liverpool's" not (new south wales* or nsw)) or ((london not (ontario* or ont or toronto*)) or ("london's" not (ontario* or ont or toronto*)) or manchester or "manchester's" or (newcastle not (new south wales* or nsw)) or ("newcastle's" not (new south wales* or nsw)) or norwich or "norwich's" or nottingham or "nottingham's" or oxford or "oxford's" or peterborough or "peterborough's" or plymouth or "plymouth's" or portsmouth or "portsmouth's" or preston or "preston's" or ripon or "ripon's" or salford or "salford's" or salisbury or "salisbury's" or sheffield or "sheffield's" or southampton or "southampton's" or st albans or stoke or "stoke's" or sunderland or "sunderland's" or truro or "truro's" or wakefield or "wakefield's" or wells or westminster or "westminster's" or winchester or "winchester's" or wolverhampton or "wolverhampton's" or (worcester not (massachusetts* or boston* or harvard*)) or ("worcester's" not (massachusetts* or boston* or harvard*)) or (york not ("new york*" or ny or ontario* or ont or toronto*)) or ("york's" not ("new york*" or ny or ontario* or ont or toronto*))))).ti,ab,in. 1971983

18 (bangor or "bangor's" or cardiff or "cardiff's" or newport or "newport's" or st asaph or "st asaph's" or st davids or swansea or "swansea's").ti,ab,in. 80317

19 (aberdeen or "aberdeen's" or dundee or "dundee's" or edinburgh or "edinburgh's" or glasgow or "glasgow's" or inverness or (perth not australia*) or ("perth's" not australia*) or stirling or "stirling's").ti,ab,in. 289930

20 (armagh or "armagh's" or belfast or "belfast's" or lisburn or "lisburn's" or londonderry or "londonderry's" or derry or "derry's" or newry or "newry's").ti,ab,in. 39086

21 or/13-20 3471084

22 (exp africa/ or exp americas/ or exp antarctic regions/ or exp arctic regions/ or exp asia/ or exp australia/ or exp oceania/) not (exp United Kingdom/ or europe/) 3605085

23 21 not 22 3250134

24 3 and 10 and 11 and 23 404

25 (Understanding the experiences of receiving and providing maternity care for autistic adults).m_titl. 1

26 Autistic Women's Experiences of the Perinatal Period A Systematic Mixed Methods Review.m_titl. 0

27 "An online survey of Autistic people's experience of care for pregnancy loss (perinatal loss) in the United Kingdom".mp. 1

28 (How do maternity services support autistic women and birthing people now What improvements could be made to help autistic people who are pregnant and giving birth and the staff who support them).m_titl. 0

29 Pregnancy outcomes in women with autism a nationwide population-based cohort study.m_titl. 1

30 or/25-29 3

31 24 and 30 3

32 3 and 10 and 23 1067

33 32 not 24 663
