# Supplementary material for: Understanding maternity care provision and experience for autistic women and birthing people and their care providers in the UK: protocol for a scoping review
Source: BMJ Open. 2026 Jul 28;16(7):e114558. doi: 10.1136/bmjopen-2025-114558 (PMC13423141; doi:10.1136/bmjopen-2025-114558)
Supplement: online supplemental file 3 [file bmjopen-16-7-s003.pdf]

Supplementary File 3. PRISMA 2020 flow diagram for new systematic reviews which included searches of databases and registers only

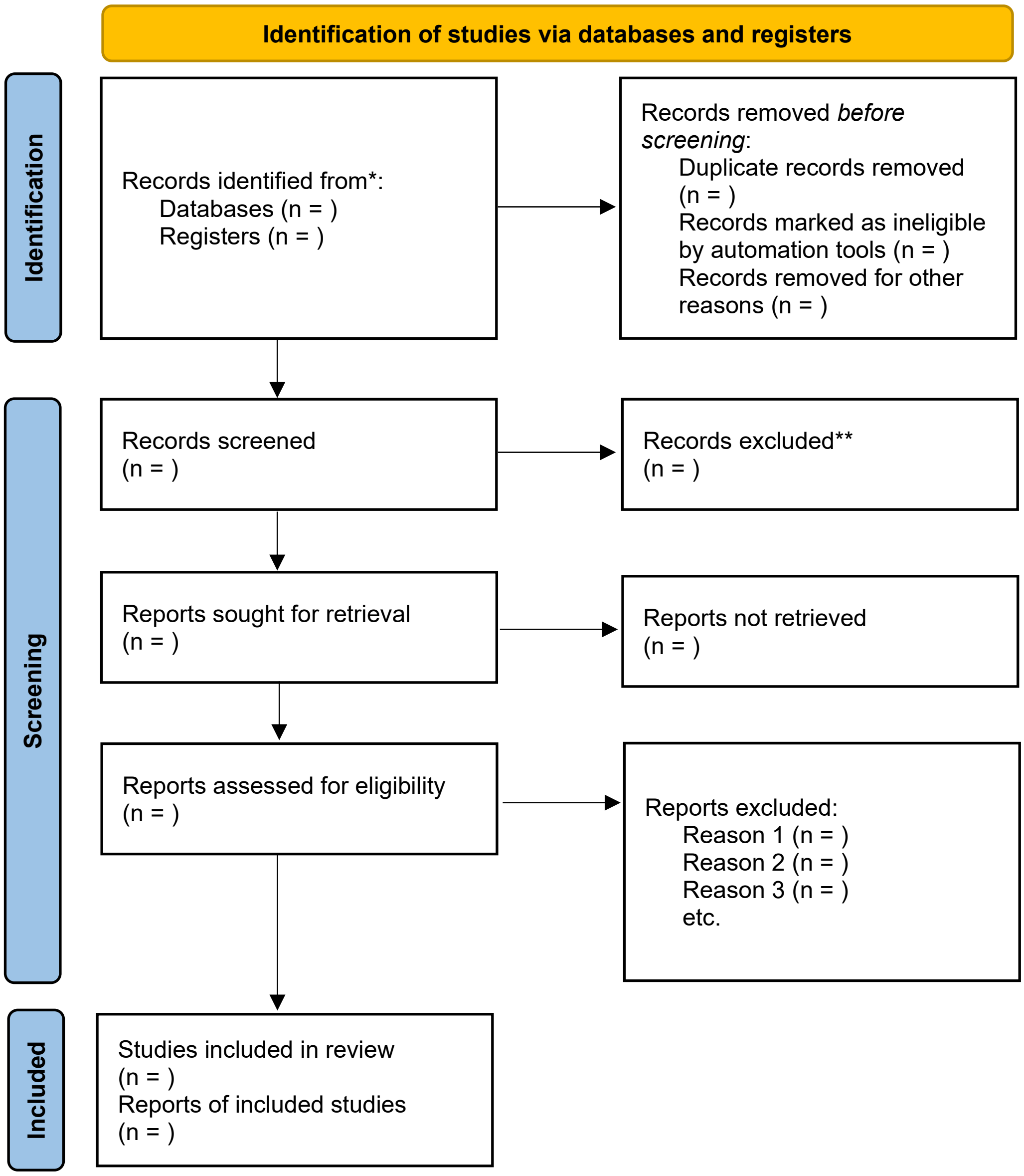

*Consider, if feasible to do so, reporting the number of records identified from each database or register searched (rather than the total number across all databases/registers).

**If automation tools were used, indicate how many records were excluded by a human and how many were excluded by automation tools.

Source: Page MJ, et al. BMJ 2021;372:n71. doi: 10.1136/bmj.n71.

This work is licensed under CC BY 4.0. To view a copy of this license, visit https://creativecommons.org/licenses/by/4.0/
