# Supplementary material for: Understanding maternity care provision and experience for autistic women and birthing people and their care providers in the UK: protocol for a scoping review
Source: BMJ Open. 2026 Jul 28;16(7):e114558. doi: 10.1136/bmjopen-2025-114558 (PMC13423141; doi:10.1136/bmjopen-2025-114558)
Supplement: online supplemental file 4 [file bmjopen-16-7-s004.pdf]

Supplementary File 4

Citation	Study Type	Setting	Population Focus	Aims/ Objectives	Methodology	Key Findings	Barriers Identified	Facilitators/ Interventions	Healthcare Provider Knowledge	Healthcare Provider Perspective	Policy/Guideline Influence	Limitations	Relevance to review questions	Notes
