# Supplementary material for: Understanding maternity care provision and experience for autistic women and birthing people and their care providers in the UK: protocol for a scoping review
Source: BMJ Open. 2026 Jul 28;16(7):e114558. doi: 10.1136/bmjopen-2025-114558 (PMC13423141; doi:10.1136/bmjopen-2025-114558)
Supplement: Supplementary file 5 [file bmjopen-2025-114558.reviewer_comments.pdf]

PEER REVIEW HISTORY

BMJ Open publishes all reviews undertaken for accepted manuscripts. Reviewers are asked to complete a checklist review form and are provided with free text boxes to elaborate on their assessment. These free text comments are reproduced below.

ARTICLE DETAILS

Title (Provisional)

Understanding maternity care provision and experience for autistic women and birthing people and their care providers in the UK: protocol for a scoping review

Authors

Welsh, Joanne; Redmond, Kathryn; McPeake, Joanne; Nikolić, Nik; Moller, Ann-Beth

---

VERSION 1 - REVIEW

Reviewer 1

Name Sathe, Nila

Affiliation RTI International, Research Triangle Park, NC, USA; RTI International-University of North Carolina Evidence-Based Practice Center US Cochrane Affiliate, Research Triangle Park, NC, USA.

Date 07-May-2026

COI None

This protocol describes an interesting scoping review addressing maternity care for people with ASD. The design and approach are generally well described, but the protocol would benefit from clarifications in a few areas:

-The relationship of KQ2 to the other KQ and overall objective is not immediately clear. I believe the outcomes you've selected to focus on will help elucidate how maternity care is provided to this population, but a more explicit linkage of that KQ to the objective would benefit readers.

-Explain inclusion/exclusion criteria in more detail. While you do not use a PICOTS framework for this scoping review, clearer explanation of study designs (e.g., presumably qualitative and quantitative) to be included would be helpful. English language publications only?

-Explain if screening and data extraction or dually performed and/or the level of QC used. You note 3 reviewers and piloting extraction but it's not clear if screening or extraction are dual.

-Is Overton the only source of grey literature? The paper would benefit from more explanation of grey literature sources and rationale for selection.

-Clarify if you will be extracting actual outcome data or counts or ...? You provide an example extraction table but it is not clear what kinds of data will be included and how synthesized.

-The abstract and background mentions provider knowledge of ASD, but the protocol does not describe capturing that as a focus or outcome. Clarify how/where this will be captured.

These clarifications should help to strengthen this protocol and clarify the approach to this useful work for readers.

VERSION 1 - AUTHOR RESPONSE

Reviewer comments	Authors responses
This protocol describes an interesting scoping review addressing maternity care for people with ASD. The design and approach are generally well described, but the protocol would benefit from clarifications in a few areas.	Thank you for these positive comments.
The relationship of KQ2 to the other KQ and overall objective is not immediately clear. I believe the outcomes you've selected to focus on will help elucidate how maternity care is provided to this population, but a more explicit linkage of that KQ to the objective would benefit readers.	Thank you for this very valid comment. We have reviewed the KQ2 and are in agreement with you that this is not within the aim of the research. We have therefore decided to remove KQ2.
Explain inclusion/exclusion criteria in more detail. While you do not use a PICOTS framework for this scoping review, clearer explanation of study designs (e.g., presumably qualitative and quantitative) to be included would be helpful. English language publications only?	Thank you for this comment. We have used the PCC framework in line with the Joanna Briggs Institute methodology for scoping reviews.<br><br>On page 7 we have clarified that we will include qualitative and quantitative studies.<br><br>On page 9 we have included details in the exclusion criteria regarding studies not published in the English language.
Explain if screening and data extraction or dually performed and/or the level of QC	Thank you for this important comment. We have clarified that piloting extraction,

used. You note 3 reviewers and piloting extraction but it's not clear if screening or extraction are dual.	screening and final extraction will be completed independently by the three reviewers. The reviewers will then meet to discuss any inconsistencies and a fourth reviewer consulted in the event of unresolvable disagreements.
Is Overton the only source of grey literature? The paper would benefit from more explanation of grey literature sources and rationale for selection.	Thank you for this important comment. We have provided further detail about the Overton Index to demonstrate the rationale for the decision to use this.
Clarify if you will be extracting actual outcome data or counts or ...? You provide an example extraction table but it is not clear what kinds of data will be included and how synthesized.	Thank you for this comment which relates to your earlier comment related to KQ2. We have revised our research questions in line with your comments and therefore will no longer be reporting on outcome data.<br><br>In terms of data related to the other research questions, we will extract key themes and summarise the findings using narrative as is typical of a scoping review.
The abstract and background mentions provider knowledge of ASD, but the protocol does not describe capturing that as a focus or outcome. Clarify how/where this will be captured.	Thank you for identifying this error. We have addressed this by adding details about knowledge of ASD to the research questions and manuscript in the appropriate areas.

VERSION 2 - REVIEW

Reviewer 1

Name Sathe, Nila

Affiliation RTI International, Research Triangle Park, NC, USA; RTI International-University of North Carolina Evidence-Based Practice Center US Cochrane Affiliate, Research Triangle Park, NC, USA.

Date 21-Jun-2026

COI

Thanks for the clarifying revisions to this protocol. The KQ changes seem to align better with your intended purpose, but I think the new KQ2 could benefit from further minor revision to align it better with the focus in the introduction--i.e., providers' knowledge about autism specifically as it relates to maternity care (e.g., supports an autistic person may need at the

time of birth). The question may benefit from further specification to make the focus immediately clear from the KQ alone (e.g., What knowledge do HCP providing maternity care have about the effects of autism on the provision of care for autistic women and birthing people?)

Additionally, I think the Overton note and limitation statement re grey literature is useful. I am not sure the extent to which literature from advocacy organization sites (such as the National Autistic Society) is indexed in Overton or indeed if relevant organizations have white papers or other materials addressing maternity care, but you've appropriately acknowledged the targeted focus of the grey literature search.

I did not see a revised supplemental file (may have missed it), but I assume it's been adjusted as needed to the reflect the KQ changes.

Thanks again for your clarifications!

VERSION 2 - AUTHOR RESPONSE

Reviewer comments	Authors responses
Thanks for the clarifying revisions to this protocol. The KQ changes seem to align better with your intended purpose, but I think the new KQ2 could benefit from further minor revision to align it better with the focus in the introduction--i.e., providers' knowledge about autism specifically as it relates to maternity care (e.g., supports an autistic person may need at the time of birth). The question may benefit from further specification to make the focus immediately clear from the KQ alone (e.g., What knowledge do HCP providing maternity care have about the effects of autism on the provision of care for autistic women and birthing people?)	Thank you for this valuable comment. We have strengthened KQ2 according to your recommendation. We have also clarified this in Table 1 – Inclusion criteria.
Additionally, I think the Overton note and limitation statement re grey literature is useful. I am not sure the extent to which literature from advocacy organization sites (such as the National Autistic Society) is indexed in Overton or indeed if relevant organizations have white papers or other materials addressing maternity care, but you've appropriately	Thank you for this comment.

acknowledged the targeted focus of the grey literature search.	
I did not see a revised supplemental file (may have missed it), but I assume it's been adjusted as needed to the reflect the KQ changes.	Apologies that you could not see this. We have resubmitted it again with this submission.

VERSION 3 - REVIEW

Reviewer 1

Name Sathe, Nila

Affiliation RTI International, Research Triangle Park, NC, USA; RTI International-University of North Carolina Evidence-Based Practice Center US Cochrane Affiliate, Research Triangle Park, NC, USA.

Date 11-Jul-2026

COI

Thank you for addressing the minor comments. I think the final review will be a useful contribution to the literature.
